# Pancreatic Injury in Severe SARS-CoV-2 Infection: A Retrospective Study Across Three Pandemic Waves

**DOI:** 10.3390/life15091439

**Published:** 2025-09-14

**Authors:** Mihai Lazar, Cristina Emilia Chitu, Ecaterina Constanta Barbu

**Affiliations:** 1 Faculty of Medicine, University of Medicine and Pharmacy Carol Davila, No. 37, Dionisie Lupu Street, Sector 2, 020021 Bucharest, Romania; mihai.lazar@umfcd.ro (M.L.); ecaterina.barbu@umfcd.ro (E.C.B.); 2 National Institute for Infectious Diseases “Prof. Dr. Matei Bals”, No. 1, Calistrat Grozovici Street, Sector 2, 021105 Bucharest, Romania

**Keywords:** SARS-CoV-2, COVID-19, pancreatitis, pancreatic injury, systemic inflammation

## Abstract

Acute pancreatitis (AP) has emerged as a notable complication in patients with COVID-19, yet the interplay between viral infection, systemic inflammation, and pancreatic injury remains incompletely understood. This study aimed to evaluatethe characteristics and risk factors of APin patients with severe COVID-19 pneumonia. We conducted a retrospective, single-center analysis of 405 hospitalized COVID-19 patients with and without AP. Laboratory markers, including CRP, ESR, fibrinogen, LDH, D-dimers, WBC, neutrophils, serum potassium, and serum glucose, alongside imaging and clinical parameters, were analyzed for associations with AP occurrence. Our results indicate that elevated inflammatory and coagulation markers, leukocytosis with neutrophilia, hyperglycemia, hypokalemia, and more severe pulmonary involvement were significantly associated with AP in COVID-19. LDH and inflammatory markers demonstrated particularly strong predictive value, while D-dimers and lung injury severity also contributed to risk stratification. These findings suggest that systemic inflammation, endothelial dysfunction, immunothrombosis, and metabolic impairments converge to increase pancreatic vulnerability in COVID-19 patients. Early recognition of these risk factors may guide monitoring and therapeutic interventions, although prospective validation is needed.

## 1. Introduction

Acute pancreatitis (AP) may be caused by multiple factors, including gallstones, alcohol consumption, hypertriglyceridemia, endoscopic retrograde cholangiopancreatography, hypercalcemia, autoimmune disorders, genetic factors, and certain medications [1,2]. Additionally, AP may be triggered by infectious agents (viral, bacterial, or parasitic), though this remains uncommon in clinical practice and is often underdiagnosed due to limited research in the area [3]. A comprehensive systematic review by Imam et al. found that viruses accounted for 65.3% of infectious etiology AP cases, with virus-attributed AP exhibiting substantially higher mortality (21.8%) compared to other infectious causes (7.0%) [4]. In a more focused review examining AP in the setting of viral hepatitis, Panic et al. reported that among 73 documented cases, the most frequently implicated agents were hepatitis A (42.5%) and E (28.8%) viruses; notably, 32.9% of cases were severe, and overall mortality reached 21.9% [5]. The main viruses associated with viral AP to date include Epstein–Barr virus, cytomegalovirus, varicella-zoster virus, measles virus, mumps virus, hepatitis viruses (A, B, C, D, E), coxsackievirus, echovirus, and human immunodeficiency virus [6,7]. More recently, SARS-CoV-2 has been recognized as a novel cause of pancreatic injury, highlighting the need for heightened clinical awareness [8,9].

Since the emergence of severe acute respiratory syndrome coronavirus 2 (SARS-CoV-2) in late 2019, the primary focus of clinical research has been on its respiratory manifestations and associated systemic inflammatory responses. However, the rapid accumulation of clinical data has revealed that coronavirus disease 2019 (COVID-19) is a multisystemic disorder, also associated with gastrointestinal and hepatobiliary involvement [10,11,12]. SARS-CoV-2 exhibits wide tissue tropism, facilitated by its capacity to bind to multiple host cell surface receptors—including angiotensin-converting enzyme 2 (ACE2), neuropilin-1, the tyrosine-protein kinase receptor UFO (AXL), and antibody–FcγR complexes—which can contribute to a spectrum of complications affecting the respiratory, cardio-vascular, gastrointestinal, and hematologic systems [13].

Among the extrapulmonary complications, acute pancreatitis (AP) has attracted growing attention, both for its clinical impact in critically ill patients and for the uncertainty surrounding its pathophysiological mechanisms.

The potential link between SARS-CoV-2 infection and AP remains a subject of intense debate. Some authors have suggested that SARS-CoV-2 may directly infect pancreatic acinar and islet cells via ACE2 receptors, which are highly expressed in pancreatic tissue [14,15]. This hypothesis is supported by reports of elevated pancreatic enzymes and imaging-confirmed AP in COVID-19 patients without traditional risk factors [16]. Conversely, other studies argue that AP in COVID-19 patients is more likely secondary to systemic inflammation, microvascular injury, or drug-induced toxicity rather than direct viral cytopathic effects [17,18].

Adding to the controversy, incidence estimates vary widely—from rare case reports to suggestions of a clinically meaningful association—likely reflecting differences in diagnostic criteria, timing of testing, and patient populations studied [19,20]. Furthermore, the effect of evolving viral variants, changes in treatment protocols, and vaccination status across successive pandemic waves on the incidence and severity of AP in COVID-19 patients remains poorly understood.

In this context, our retrospective analyses that span multiple phases of the pandemic can help characterize the involvement of viral factors, host responses, and therapeutic interventions in the development of AP. In our research, we present a multi-wave retrospective comparative study evaluating the occurrence, characteristics, and risk factors of AP in severe SARS-CoV-2 infection, providing new insights into a still-unresolved clinical question.

## 2. Materials and Methods

### 2.1. Study Population

We conducted a retrospective, observational cohort study at a single tertiary care center dedicated exclusively to the management of COVID-19 patients. The study included 405 individuals with severe COVID-19 admitted between March 2020 and December 2023, stratified into three cohorts according to their admission period, reflecting the first three pandemic waves: 135 patients (March 2020–January 2021), 135 patients (February 2021–June 2021), and 135 patients (July 2021–December 2021).We further divided the patients into two groups: Group A (32 patients with AP) and Group B (373 patients without AP).

Eligibility criteria included adult patients (≥18 years) with confirmed SARS-CoV-2 infection, established either by real-time polymerase chain reaction (RT-PCR) or rapid antigen testing, who presented with severe disease and underwent CT scanning upon admission. Only patients with a CT image quality score of 4 or 5 were considered.

Exclusion criteria included the following: (a) age under 18 years; (b) pregnancy; (c) pre-existing chronic pancreatic disorders; (d) pancreatic malignancy; (e) CT image quality score between 1 and 3; (f) pancreas not included/only partially included in the thoracic CT scan; (g) documented iodine contrast intolerance.

**Figure 1:**
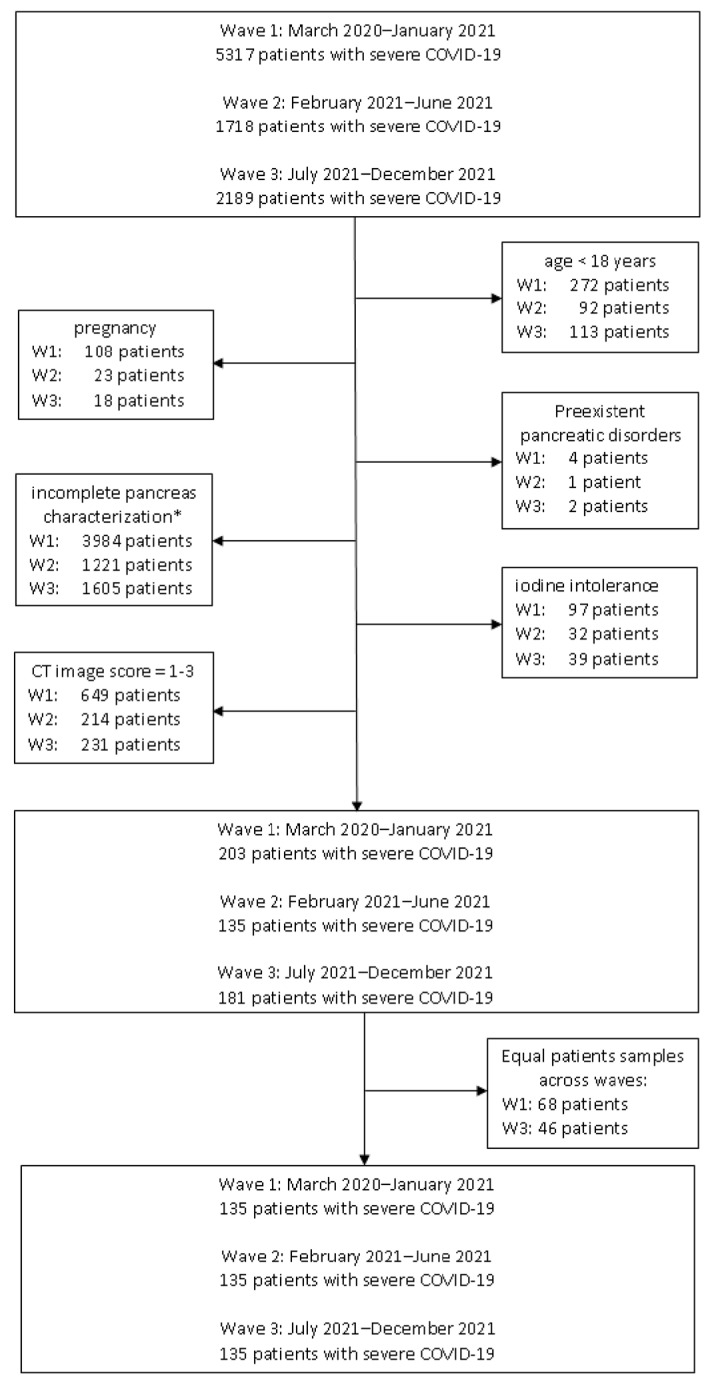
Flow diagram of patient selection. Abbreviations: W1—wave1; W2—wave 2; W3—wave 3. * The pancreas was not included/was only partially included in the thoracic CT scan, or the CT scan was performed without intravenous iodine contrast, which resulted in incomplete pancreas characterization.

The flowdiagram regarding the patient selection is presented in Figure 1.

### 2.2. Definitions

We considered a severe form of COVID-19 when the patients presented at least one of the following criteria: peripheral oxygen saturation (SpO_2_) ≤93% in the ambient air, respiratory rate (RR) >30/min, arterial oxygen partial pressure to fractional inspired oxygen ratio (PaO_2_/FiO_2_ ratio) <300 or lung infiltrates >50% of lung parenchyma [21].

The overall quality of each CT scan was assessed based on the clarity of lung parenchyma visualization, the sharpness of vascular anatomical contours, and the degree of motion artifacts related to respiration or patient movement, using the following grading scale:

1. Poor—indistinct parenchymal detail, poorly defined contours, and pronounced motion artifacts.
2. Fair—acceptable parenchymal clarity and contour definition, with moderate motion artifacts.
3. Adequate—acceptable clarity and contour definition, with occasional motion artifacts.
4. Good—well-defined parenchymal structures and contours, with minimal motion artifacts.
5. Excellent—sharply defined parenchymal and vascular anatomy, with no motion artifacts [22].

For positive diagnosis of AP, we used the Revised Atlanta Classification (2012), considering a positive diagnosis if two of the following three criteria were met:

- clinical
  - sudden onset of epigastric pain, often radiating to the back;
  - nausea, vomiting;
- serum amylase or lipase ≥ 3× upper limit of normal (ULN)—we used the pancreatic lipase in our study because it is more specific and remains elevated longer;
- contrastenhanced CT (CECT)—for assessing severity and complications [23].

The severity of AP was evaluated by the Balthazar score/CTSI (CT severity index), which characterizes the severity of AP by CT scan, combining the pancreatic morphologic modifications and the pancreatic necrosis [24].

Balthazar Grade (0–4 points):

- normal pancreas → 0;
- diffuse or focal enlargement of the pancreas → 1;
- minimal peripancreatic changes → 2;
- single peripancreatic fluid collection → 3;
- ≥2 fluid collections or presence of peripancreatic gas → 4.

Pancreatic necrosis score (0–6 points):

- 0%→ 0;
- ≤30%→ 2;
- 30–50%→ 4;
- 50%→ 6.

Final CTSI score = Balthazar grade + pancreatic necrosis score (maximum 10 points):

- 0–3: mild AP;
- 4–6: moderate;
- 7–10: severe, high risk of complications/mortality.

We divided the patients into 3 categories: mild AP (score from 0 to 3), moderate AP (score from 4 to 6), and severe AP (score from 7 to 10).

### 2.3. Demographic and Biological Parameters

For each patient enrolled in the study, we collected demographic data (sex, age), clinical variables (heart rate, respiratory rate, peripheral oxygen saturation, length of hospitalization), inflammatory markers [C-reactive protein (CRP), fibrinogen, serum ferritin, erythrocyte sedimentation rate (ESR), interleukin-1 (IL-1), interleukin-6 (IL-6), tumor necrosis factor alpha (TNF-α)], biochemical parameters (alanine aminotransferase, creatine kinase, lactate dehydrogenase, lipase, urea, creatinine, conjugated and unconjugated bilirubin, blood glucose), complete blood count (erythrocytes, leukocytes, lymphocytes, neutrophils), D-dimers levels, and serum electrolytes (sodium, potassium).

### 2.4. CT Examination Protocol

At hospital admission, all patients underwent chest CT scanning using a 64-slice Definition AS system (Siemens Healthcare GmbH, Munich, Germany). Imaging was performed in helical mode with CAREDose4D and CARE kV enabled to minimize radiation exposure.

The acquisition parameters are presented in Table 1.

Quantitative assessment of lung lesions was performed using the *syngoPulmo3D* software (version VA48A), which enabled calculation of lesion percentage and volume within predefined density thresholds. Lung lesions were divided into alveolar lesion—the lung areas with densities higher than 0 Hounsfield units (HU); mixt lesions (alveolar and interstitial)—the lung areas with densities between 0 and −200 HU; interstitial lesions—the lung areas withdensities between −200 and −800 HU; and normal parenchyma—the lung areas with densities between −800 and −1000 HU [13,21]. Image analysis was carried out in a blinded fashion: the interpreting radiologist had no access to patient identifiers, study group allocation, or biological test results. All patient data were anonymized for analysis.

### 2.5. Identification of the Risk Factors/Predictors for AP in Patients with SARS-CoV-2 Infection

We used the Mann–Whitney test to observe the variations with statistical significance between Group A and B for continuous variables, while for nominal variables we applied univariate logistic regression. Associations between AP and admission variables were explored using Spearman’s rank correlation. To further assess discriminatory ability for each risk factor of AP, we used ROC curve analysis. Variables meeting statistical or clinical relevance criteria were entered into multivariable logistic regression to construct prognostic models for AP.

### 2.6. Statistical Analysis

Statistical analyses were conducted using SPSS version 25 (IBM Corp., Armonk, NY, USA). Continuous variables are expressed as medians with interquartile ranges, and categorical variables as percentages. Differences among the three groups were evaluated by the Mann-Whitney U test and binary logistic regression. Associations with AP were examined for all parameters using Spearman’s correlation and ROC curve analyses to further assess predictive accuracy. Variables with clinical or statistical relevance were entered into multivariable logistic regression models developed via backward elimination, excluding predictors with *p* > 0.2 based on Wald statistics. Model fit was assessed by the Omnibus test, with *p* < 0.05 denoting statistical significance [22].

## 3. Results

We found AP in 32 cases, representing approximately 7.9% of the total patients included in the study, with a median age of 62 [56.2; 69.5] years and a male/female ratio of 1.9:1, compared to a median age of 51 [41.5; 62] years and a male/female ratio of 1.5:1 for the patients without AP.

Group A patients exhibited a higher prevalence of comorbidities—including arterial hypertension, obesity, and type 2 diabetes mellitus—compared with those in Group B (Table 2). However, no statistically significant association was observed between any individual comorbidity and the occurrence of AP.

The patients with AP presented a longer hospital stay, necessitated a longer antiviral and corticoid treatment, showed a higher plasmatic glucose, a more pronounced inflammatory syndrome (illustrated by increases in CRP, ESR, and fibrinogen), cytolytic syndrome (higher serum LDH and AST), higher leukocytes and neutrophils values, mild increases in platelets, D-Dimers, potassium, and a more severe lung involvement demonstrated by higher percentages of mixed and interstitial lung lesions (Table 3).

We found pleural effusion in 122 patients (30.1%): 18 cases (56.2%) in Group A (ranging from 4 to 24 mm) and 104 cases (27.8%) in Group B (ranging from 3 to 75 mm). Pericardial effusions were found in 91 patients (22.4%): 11 cases (34.3%) in Group A (ranging from 4 to 24 mm) and 80 cases (21.4%) in Group B (ranging from 3 to 75 mm).

The overall mortality in the study was 25.7% (104 patients), with a higher percentage in Group A (37.5%) compared with Group B (24.7%). Although mortality was higher in Group A, logistic regression analysis showed that AP was not an independent risk factor for mortality (*p* = 0.115; 95% C.I. = (0.863: 3.893)). The higher mortality in Group A was instead attributable to more severe forms of COVID-19. This finding also correlates with the fact that Group A included mostly cases of mild pancreatitis, with no patients presenting with severe forms (Table 4).

The number of patients presenting pancreatic inflammation was similar across the three waves, with a slightly higher number in wave 3; the patients in wave 3 also developed a more pronounced inflammatory process compared to the first two waves.

**Figure 2:**
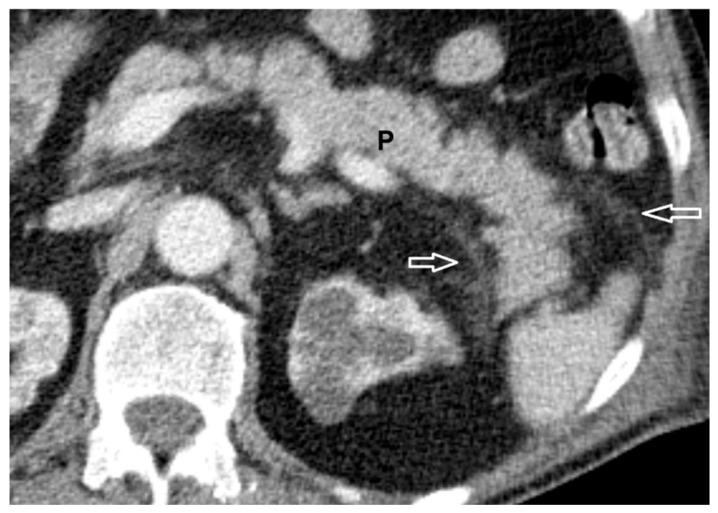
Mild AP—reduced iodine contrast uptake of pancreas (P) and mild inflammatory changes in the peripancreatic fat (white arrows).

**Figure 3:**
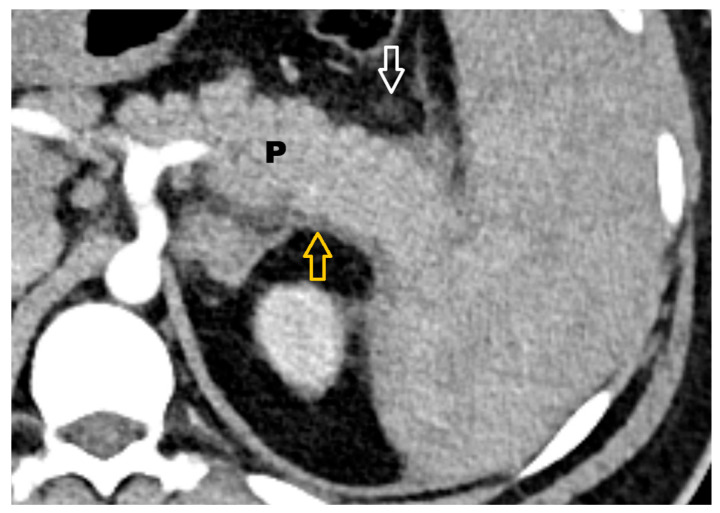
Moderate AP—enlarged pancreas (P) with reduced iodine contrast uptake, mild inflammatory changes in the peripancreatic fat (white arrow), and peripancreatic fluid collection (orange arrow).

In Group A, 29 patients (90.6%) presented with mild AP (Figure 2). Moderate forms of AP (Figure 3) were observed in only three cases—one patient during the first wave and two patients during the third wave. No cases of severe AP were recorded.

The occurrence of AP presented the highest proportional correlation with LDH and ESR, followed by the severity of lung involvement (total pulmonary lesions and interstitial lesions) (Table 5).

**Figure 4:**
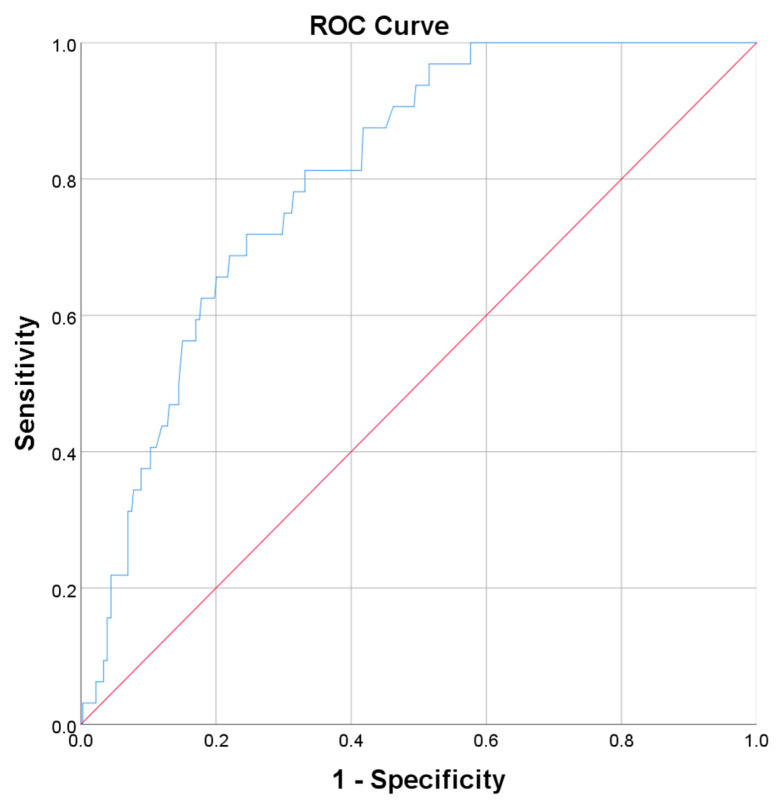
Receiver operating characteristics (ROC) curve for the ability of the “LDH” to predict pancreatitis.

We performed ROC curves analysis for all parameters presented in Table 5, which presented a significant association with AP, to further evaluate their performance in predicting the risk of AP.The highest AUC value was registered for LDH (Figure 4), followed by ESR and the percent of interstitial lung lesions (Table 6).

We performed a further multivariable logistic regression analysis using the variables characterized in Table 5 and Table 6 to identify a prediction model for AP in patients with SARS-CoV-2 infection. The multivariable logistic regression model is presented in Table 7. The statistical significance of the multivariable logistic regression estimated by the Omnibus test of model coefficients was lower than 0.001, with an overall accuracy prediction of 89.8%.

Based on the data in Table 7, we can also calculate the probability of AP in patients with SARS-CoV-2 infection, using the following exploratory formula:

EXP (−5.552 + 0.002 × D-Dimers + 0.045× Lung interstitial lesions + 0.011 × CRP)/ [1 + EXP (−5.552 + 0.002 × D-Dimers + 0.045 × Lung interstitial lesions + 0.011 × CRP)]

## 4. Discussion

### 4.1. Epidemiology and Demographics

In the general population of hospitalized COVID-19 patients, acute pancreatitis is relatively rare. A large U.S. hospital cohort of 48,012 admissions, of whom 11,883 tested positive for SARS-CoV-2, found that 32 COVID-19 patients developed AP—yielding a point prevalence of 0.27%among hospitalized COVID-19 cases [25]. In critically ill individuals, particularly those admitted to intensive care units (ICUs), SARS-CoV-2 infection conferred a significantly increased likelihood of pancreatitis, with one ICU study reporting that COVID-19 patients were5.4 times more likely to develop AP than their non-COVID counterparts (OR = 5.42; 95% CI: 2.35–6.58) [26]. The reported incidence of AP in SARS-CoV-2 infection shows variations of 0.3% and 17% between studies [16,27,28], largely depending on the inclusion criteria of the patients and the study type; therefore, the incidence of AP in our study (7.9%) may be explained by the severe COVID-19 cases enrolled.

The prospective, international COVID-PAN collaborative cohort (n = 1777 AP admissions from March–July 2020) found that concomitant SARS-CoV-2 infection was associated with higher odds of AP, organ failure, longer hospital stay, and increased 30-day mortality (14.7% vs. 2.6%) compared with AP without SARS-CoV-2, even after multilevel adjustment [29].

The age, incidence, and mortality in patients with SARS-CoV-2 infection associated with AP show large variations between authors. Bulthuis et al. found that COVID-19-attributed AP cases have a median age of60 (range 47–71), with 80% men, and a mortality of 60% [30]. Vatansev et al. communicated a similar sex ratio (M : F ratio of 1.6/1) and age of 62.22 ± 16.37, associated with a mortality of 8% in patients without AP and 28% in patients with AP [31]. In a study conducted by Chaudry et al., males accounted for 54.04% of the patients with COVID-19 and AP.The mortality rate in both patients with AP and without was 13.39%, with significantly higher mortality observed in patients with AP on multivariate analysis (OR: 1.19) [27]. In our study, we registered a higher male–female sex ratio (1.9:1 vs. 1:5) and a higher median age (62 vs. 51 years) in patients with AP and COVID-19, suggesting a higher risk for older males to have both conditions. We found a mortality of 37.5% in patients with AP and COVID-19, compared to 25.7% in patients without AP, indicating a more severe outcome in the case of AP. This investigation was conducted at a single tertiary-care COVID-19 center. While this setting provided a homogeneous patient population and standardized diagnostic protocols, it inherently limits generalizability. Patient demographics, treatment strategies, and thresholds for diagnostic imaging may differ in other institutions and healthcare systems.

### 4.2. Pathophysiological Mechanisms

#### 4.2.1. DirectViral Injury Can Induce AP

SARS-CoV-2 gains entry into cells via the ACE2 receptor, primed by the transmembrane serine protease 2(TMPRSS2); importantly, both are expressed in pancreatic ductal, acinar, and islet cells, enabling viral tropism beyond the respiratory tract, with transmembrane serine protease 2(TMPRSS2) expression being mainly present in ductal cells, and a limited co-expression with ACE2 in beta cells [32,33].

In human pancreatic organ cultures and autopsy tissue, SARS-CoV-2 infected both endocrine and exocrine cells and replicated in situ, establishing cellular permissiveness beyond the islet compartment [34]. Single-cell analyses of infected primary human pancreas samples showed that infection is ACE2-dependent and can target multiple pancreatic cell types; while the extent of infection was circumscribed and often non-lytic, the very presence of viral transcripts/proteins within exocrine lineages confirms direct tropism [35].

ACE2–spike binding triggers internalization of the receptor–virus complex; TMPRSS2 cleavage favors rapid entry, whereas ADAM17 and other proteases can remove ACE2 ectodomains. Furthermore, surface ACE2 is depleted, diminishing its enzymatic counterbalance to Ang II. The resulting Ang II accumulation amplifies AT1R signaling (NADPH oxidase-driven ROS, NF-κB activation, and microvascular constriction) and fuels sterile inflammation, a known amplifier of pancreatic injury [36,37]. Both theoretical and experimental studies involving other organs provide support for this mechanism; in the pancreas, the consequence would be vasoconstriction and capillary no-reflow within lobules, potentiating acinar ischemia and necrosis once infection is established [36,37]. Single-cell and histologic analyses showed SARS-CoV-2 presence in ductal, endothelial, and islet cells of the pancreas associated with generalized fibrosis and multiple vascular thrombi, suggesting that SARS-CoV-2 infection of the pancreas may promote acute and chronic pancreatic dysfunction [38]. Acinar cells are susceptible to endoplasmic reticulum (ER)-stress/autophagy derailment. Thus, it can precipitate zymogen activation and promote necrosis with pancreatic and peripancreatic injury [37]. Ductal cells mediate bicarbonate-rich fluid secretion, regulate luminal pH, and coordinate enzyme flushing from acini to the duodenum. Infection-induced ductal epithelial injury can reduce bicarbonate output, increase ductal viscosity, and promote protein plug formation—conditions that favor intraductal trypsinogen activation and secondary acinar injury. Experimental infection of ductal epithelium (in non-human primates (NHPs) and human tissue) strengthens this mechanistic link between direct cytopathic injury and the classic obstructive–secretory model of AP [38]. Pancreatic β-cell infection can impair insulin secretion and promote apoptosis, creating local metabolic stress that aggravates acinar susceptibility to cytokine injury. Conversely, IL-1β and monocyte chemoattractant protein-1 (MCP-1) from infiltrating myeloid cells worsen β-cell dysfunction, creating a paracrine loop between endocrine and exocrine compartments [35,39,40].

#### 4.2.2. Systemic COVID-19 Inflammation as a Trigger for Pancreatic Injury

Severe COVID-19 is characterized by a dysregulated host response with high circulating cytokines and chemokines (a “cytokine storm” phenotype) including IL-1β, IL-6, TNF, and CXCL10/IP-10 (C–X–C motif chemokine 10 (CXCL10), also known as interferon γ-induced protein 10 kDa (IP-10)), and MCP-1/CCL2 (C-C motif ligand 2 (CCL2), also known as monocytic chemotactic protein 1 (MCP-1)), among others [41,42,43]. IL-1β is a pivotal upstream cytokine produced after assembly of the NOD-like receptor protein 3 (NLRP3) inflammasome and caspase-1 activation. Multiple lines of evidence in COVID-19 show NLRP3 priming/activation in circulating leukocytes and tissues, with increased IL-1β correlating with severity and hypercoagulability [41,42,44,45]. In AP models, NLRP3 activation in acinar and immune cells drives cleavage of pro-IL-1β and pro-IL-18, neutrophil influx, and tissue injury; genetic or pharmacologic attenuation of NLRP3 reduces pancreatic necro-inflammation and lung injury [46,47,48]. In COVID-19, multiple systemic triggers activate NLRP3. These include viral RNA, pathogen-associated molecular patterns (PAMPs), damage-associated molecular patterns (DAMPs) from injured tissues, hypoxemia, reactive oxygen species (ROS), and lipid-related stimuli such as crystals or fatty acids. This activation amplifies IL-1β production. IL-1β then increases endothelial adhesion molecules and vascular permeability. It also promotes trypsinogen activation and acinar cell stress responses. Finally, it induces secondary chemokine programs in pancreatic stromal and ductal cells [48,49,50,51].

IL-1β can also promote adipose inflammation and lipolysis; recruited C-C chemokine receptor 2 (CCR2+) monocytes/macrophages in peripancreatic fat amplify local cytokine production and ROS, impairing microcirculatory flow and worsening parenchymal injury [47,52]. Systemic inflammatory mediators may also activate pancreatic stellate cells, promoting fibrosis via extracellular matrix deposition and also contributing to a chronic pancreatic dysfunction [53]. IL-1 receptor agonists bind to IL-1 receptor type I (IL-1R1), preventing IL-1α and IL-1β from activating downstream inflammatory pathways; their administration may induce an increase in IL-1 plasmatic levels because the receptor blockade removes negative feedback loops, leading to sustained production [54]. Therefore, the plasmatic value of IL-1 may not adequately reflect the severity of the inflammatory process in these cases and requires correlation with other inflammatory markers.

#### 4.2.3. Drug-Induced Pancreatic Injury

The prostaglandins exerta cytoprotective effect on pancreatic acinar and ductal cells, stabilizing membranes and maintaining ductal patency; their depletion in the context of nonsteroidal anti-inflammatory drug (NSAID) administration predisposes to pancreatic duct constriction, increased permeability (facilitating enzyme retention and autodigestion), and a reduction in systemic glutathione caused by decreased superoxide dismutase activity (which prevents the appropriate response against oxidative stresses) [55,56,57,58]. Interestingly, this implies a small paradox: NSAIDs may both protect against inflammation via cyclooxygenase (COX) and phospholipase A_2_ (PLA_2_) inhibition and also promote injury via loss of prostaglandin-mediated cytoprotection.

Several reports suggest that NSAIDs (or their metabolites) are responsible for direct toxic effects on pancreatic acinar cells due to the destabilization of the cellular membrane, intracellular calcium dysregulation, and premature activation of digestive zymogens. Prostaglandin inhibition may reduce pancreatic perfusion or alter cellular secretions, predisposing to ischemia or ductal dysfunction and thus pancreatic injury. Additionally, a few systematic reviews recognize the drug-induced AP explained through immune/hypersensitivity mechanisms (eosinophilic or lymphocytic infiltration) induced by NSAIDs in susceptible individuals [59,60].

Glucocorticoids (frequently administrated in COVID-19 patients)(i) may disrupt intracellular calcium regulation in acinar cells, altering secretion dynamics and increasing susceptibility to injury; (ii) may increase viscosity of pancreatic secretions, producing protein-rich plugs in ductules that lead to localized obstruction, stasis, and acinar injury; (iii) reduce pancreatic volume as well as bicarbonate and amylase secretion, possibly sustaining a pro-injury state by maladaptive secretory suppression; (iv) may mediate an indirect metabolic route to pancreatic inflammation due to steroid-induced hyperlipidemia [61,62,63]. Interestingly, high-dose corticosteroids have been shown in animal models to reduce pancreatic inflammation and edema by inhibiting leukocyte activation and cytokine release; this highlights contextdependency and suggests that direct toxic, metabolic, or secretory mechanisms may predominate in steroid-induced AP [64].

Administration of corticosteroids and NSAIDs, particularly in patients with prolonged hospitalizations, may independently induce pancreatic inflammation regardless of the direct viral effects on the pancreas, thereby contributing to an increased incidence of acute pancreatitis in severe COVID-19 cases [59,60].

However, in the context of COVID-19 care, both steroid and antiviral treatment durations scale with disease severity and length of hospital stay. Severe systemic inflammation independently correlates with pancreatic enzyme elevations, so longer treatment can be a surrogate marker of illness severity and not a causal driver of AP. Elevated pancreatic enzymes should therefore be correlated with other severity metrics (oxygen/ventilation level, severity scores, CRP/IL-6, D-dimers), ICU exposure, and illness duration to mitigate confounding by indication. Patients requiring oxygen or ventilatory support are reported to receive dexamethasone for up to 10 days in the RECOVERY protocol; thus, longer exposure correlates with baseline severity and multisystem inflammation—each independently linked to pancreatic enzyme elevations, potentially creating confounding by severity [65].

Considering the above mechanisms, the evidence supports a streamlined, stepwise model in which SARS-CoV-2 infection induces AP:

(i) Seeding: virus reaches the pancreas via haematogenous spread or retrograde ductal flow. Ductal epithelial cells and capillary endothelium expressing ACE2 (with TMPRSS2) are initial targets.
(ii) Entry and ACE2 depletion: virus spikes–ACE2 engagement and TMPRSS2 priming permit fusion; receptor internalization and sheddase activity deplete surface ACE2, locally increasing angiotensin (Ang) II tone and reducing Ang-(1–7).
(iii) Cytopathic stress: infected ductal cells reduce bicarbonate secretion and alter luminal rheology; infected acinar cells experience ER stress and impaired autophagic flux, leading to trypsinogen activation, vacuolization, and mitochondria-dependent cell death.
(iv) Microvascular injury: endothelial infection and Ang II–biased signaling drive microthrombi and ischemia (“thrombofibrosis”), compounding acinar necrosis.
(v) Lesion propagation: DAMPs, activated proteases, and cytokines amplify lobular injury and recruit inflammatory cells, closing a feed-forward loop of AP.
(vi) NSAIDs and corticosteroids: may aggravate existing pancreatic inflammation or even induce it via prostaglandin depletion, leading to ductal changes, direct oxidative injury, secretory alteration, and hyperlipidemia.

### 4.3. Risk Factors in AP

The interplay between systemic inflammation and the development of AP in COVID-19 patients has garnered significant attention. Acute-phase reactants such as C-reactive protein (CRP), fibrinogen, and erythrocyte sedimentation rate (ESR) serve as biomarkers reflecting the inflammatory environment. Their elevated levels have been implicated in the pathogenesis of AP in the context of SARS-CoV-2 infection, as presented in Section 4.2. Although, cytokine-driven inflammation (expressed by increased blood levels of TNFα, IL-1, IL-6, ESR, fibrinogen, and CRP) and dysregulated immune responses are associated with a broad spectrum of pathologies, they are generally used to assess disease severity [66,67,68]. A study by Qian et al. demonstrated that higher CRP levels were associated with increased severity and mortality in COVID-19 patients [69]. Similarly, Ding et al. reported that elevated CRP levels correlated with increased pancreatic enzyme levels, suggesting a link between systemic inflammation and pancreatic injury in COVID-19 patients [70]. In COVID-19 patients, increased fibrinogen levels have been associated with a prothrombotic state and poor prognosis. Pawlowski et al. observed that COVID-19 patients exhibited higher fibrinogen levels compared to non-COVID-19 patients, with a significant proportion developing thromboembolic events.In the setting of AP, elevated fibrinogen levels may contribute to thrombotic complications, exacerbating pancreatic injury and leading to worse clinical outcomes [71].

Although ESR is a nonspecific marker of inflammation, and generally considered less sensitive than CRP, we found a higher AUC for elevated ESR than for CRP or fibrinogen in patients with COVID-19 and AP (0.777 vs. 0.702 and 0.675, respectively), indicating that ESR can be used as a reliable marker to assess the severity of the inflammatory process in patients with COVID-19 and AP.

Elevated serum LDH levels are indicative of tissue damage and have been associated with various inflammatory conditions. In the context of COVID-19, increased LDH levels have been generally linked to disease severity and poor prognosis. For instance, a study by Huang et al. involving 1.751 patients from Leishenshan Hospital in Wuhan, China, found that increased LDH levels were associated with a higher risk of in-hospital death, regardless of whether LDH was considered as a classified or continuous variable [72]. Evaluating LDH in SARS-CoV-2 infection, some authors have observed that patients with COVID-19 who developed AP had higher levels of LDH is indicative of severe systemic inflammation and organ dysfunction, including pancreatic injury [73,74]. In our study, LDH values were significantly higher in patients with AP and COVID-19 (381 vs. 257 UI/L), with higher LDH values indicating a higher risk of AP (AUC = 0.806, *p* < 0.001, 95% CI(0.743; 0.868)), suggesting that LDH may serve as a useful biomarker for identifying patients with COVID-19 at risk of AP.

Even before the pandemic, observational studies have demonstrated that elevated D-dimer levels correlate with disease severity and local complications in acute pancreatitis—including pancreatic necrosis, infected necrosis, persistent organ failure, and mortality—and that baseline D-dimer measurements provide incremental prognostic information beyond conventional scores [75,76]. In COVID-19 cohorts, elevated pancreatic enzymes frequently parallel rises in D-dimers; large registry and single-centre analyses linkhyperlipasemia in SARS-CoV-2 infection with higher D-dimers quartiles and worse outcomes (increased intensive care unit (ICU) utilization, mechanical ventilation, and mortality), suggesting a statistical association between coagulation activation and pancreatic biochemical perturbation [73].

Additionally, endotheliitis and alveolar-initiated immunothrombosis can generalize to systemic microthrombotic states. These can involve splanchnic and pancreatic microcirculation, leading to focal ischemia, enzymatic activation, and necrosis. Such pathways are supported by both basic and clinical studies of coagulation in acute pancreatitis, as well as by pathophysiological studies of COVID-19-associated coagulopathy [77,78]. Thus, D-dimer elevation in a COVID-19 patient with abdominal pain or hyperenzymemia should prompt a high index of suspicion for pancreatic involvement and for concurrent thrombotic complications, and warrant early multimodal assessments(cross-sectional pancreatic imaging, serial enzyme and coagulation testing, and consideration of thromboembolic workup) [75,79]. Prospective validation is required to determine whether D-dimersare causally implicated in pancreatic injury during SARS-CoV-2 infection (mediator) or serve primarily as a disease-severity marker (confounder). Randomized trials of anticoagulation in COVID-19 have shown mixed effects on organ-specific outcomes, and dedicated studies are needed to evaluate whether antithrombotic interventions mitigate pancreatic necrosis or improve AP-related endpoints in infected patients [80,81]. In our study, the serum value of D-dimers was significantly higher in patients with AP, with the ROC curve analysis indicating a good association and an independent predictive value for D-dimers in the context of AP (AUC = 0.684). Therefore, we considered that the integration of D-dimers into multimodal risk stratification for COVID-19 patients with abdominal symptoms is a rational, evidence-informed step that may facilitate earlier recognition of pancreatic complications and triage to targeted supportive and interventional therapies.

Leukocytosis with neutrophilic predominance constitutes a cardinal feature of the innate immune response to both AP and severe SARS-CoV-2 infection; mounting evidence now implicates dysregulated neutrophil activation as a mechanistic connection that may increase the risk and severity of pancreatic injury in COVID-19. In AP, early neutrophil recruitment amplifies local proteolytic cascades, promotes formation of neutrophil extracellular traps (NETs), and facilitates trypsinogen activation and acinar cell injury—pathways shown experimentally to intensify parenchymal necrosis and systemic inflammation [82]. SARS-CoV-2 infection produces a stereotyped hematologic response characterized by increased WBC, neutrophilia, and lymphopenia. The augmented circulating neutrophil pool in COVID-19 is hyperactivated and prone to NETosis, releasing chromatin, histones, and proteases that are both pro-inflammatory and pro-thrombotic. NETs have been demonstrated in the circulation and affected organs of patients with severe COVID-19 and are causally implicated in microvascular occlusion and tissue ischemia [83,84]. NETs can occlude pancreatic ducts and small pancreatic microvessels, establishing focal ischemia, facilitating intra-pancreatic activation of zymogens, and amplifying sterile inflammation; experimental models showed that NET formation directly promotes trypsin activation and parenchymal injury in severe AP. Thus, in a patient with SARS-CoV-2 infection, neutrophilia and an elevated neutrophil-to-lymphocyte ratio (NLR) may not merely reflect systemic disease severity but could act as proximate effectors that predispose to or worsen AP [82,85].

Clinically, several observational and registry studies of AP and COVID-19 report that elevated WBC counts, neutrophil predominance, and higher NLRs are associated with more severe disease phenotypes, persistent organ failure, and adverse trajectories. In COVID-19 patients, when these hematologic patterns accompany biochemical evidence of pancreatic injury, they should raise concern for clinically significant pancreatitis. Such presentations should trigger immediate imaging and appropriate supportive interventions [86,87].

The data in our study indicate increased WBC and neutrophils in patients with COVID-19 and AP, with similar values in terms of lymphocyte count, similar to the data mentioned above. However, elevated WBC and neutrophils may act as confounders—surrogate markers of systemic severity rather than direct mediators of pancreatic injury—and heterogeneity in AP aetiology (gallstone, alcohol, hypertriglyceridaemia) complicates interpretation. Rigorous prospective cohorts and mechanistic studies that pair serial leukocyte phenotyping, NET quantification, and pancreatic outcome measures in SARS-CoV-2 cohorts are required to better delineate whether modulation of neutrophil activity (for example, NET-targeting therapeutics or selective anti-inflammatory strategies) can reduce AP incidence or severity in COVID-19.

Both SARS-CoV-2 infection and acute pancreatitis (AP) are associated with elevated LDH, inflammatory markers, and leukocytosis. When these conditions coexist, it becomes challenging to determine the extent to which each contributes to the elevation of these biomarkers. This is particularly relevant in severe COVID-19 complicated by AP, where the cytokine storm drives marked increases in inflammatory markers and serum lipase, thereby amplifying the risk of mortality [32]. In their meta-analysis, Hegyi et al. reported similarly elevated levels of pro-inflammatory mediators—IL-6, IL-8, and IL-10—in patients with both COVID-19 and AP, accompanied by inflammatory cell migration to sites of infection/inflammation and activation of coagulation pathways [88,89]. These processes lead to hypoperfusion and ischemia, perpetuating inflammation and establishing a vicious cycle. Consequently, it remains difficult to distinguish between severe AP induced by COVID-19 and severe AP coinciding with COVID-19 [32].

Serum potassium (serum K^+^) abnormalities are biologically plausible modulators of pancreatic injury risk and clinical trajectory in patients with COVID-19 who develop AP. SARS-CoV-2 disrupts the renin–angiotensin–aldosterone system via ACE2 downregulation, favoring kaliuresis and hypokalemia; diuretic exposure, diarrhea, and corticosteroid use further amplify these impairments in hospitalized cohorts [90,91]. Across observational studies and reviews, hypokalemia is frequent in COVID-19 and correlates with inflammatory burden and illness severity, making serum K^+^ a readily available systemic risk indicator in this setting [90,91]. In patients with concurrent COVID-19 and AP, the intersection of virus-related hypokalemia and AP-related gastrointestinal losses (vomiting, nasogastric drainage) creates a “double hit” on potassium homeostasis. This milieu heightens risks of ileus, delays in initiating or maintaining enteral feeding, and cardiac instability during aggressive fluid therapy—factors that can convert a potentially mild episode into a complicated course [92,93].

COVID-19 amplifies dysglycemia via convergent mechanisms—cytokine-driven insulin resistance, glucocorticoid exposure, and especially, direct and indirect pancreatic involvement. Clinical series and mechanistic work in SARS-CoV-2 infection show that hyperglycemia at hospital admission (even in individuals without known diabetes) had increased values, reflecting important insulin resistance and altered adipose–liver crosstalk; such stress hyperglycemia is a stronger predictor of poor outcomes than antecedent HbA1c alone [94,95].

Furthermore, expression of the viral entry receptor ACE2 in pancreatic tissue provides a biologically plausible route for islet and acinar perturbation during SARS-CoV-2 infection, with reports linking pancreatic enzyme elevations and imaging abnormalities to the infection milieu [5]. Within this context, serum glucose in patients with COVID-19 who develop AP serves as both a prognostic biomarker and a modifiable therapeutic target. First, hyperglycemia may worsen pancreatic microcirculatory dysfunction and leukocyte–endothelial interactions, amplifying necroinflammation and organ failure—mechanisms consistent with the broader COVID-19 hyperglycemia literature [94,96]. Second, COVID-19-associated AP cohorts show higher admission glucose and worse clinical trajectories than non-COVID AP comparators [97]. Third, because stress hyperglycemia is dynamic, monitoring coupled with judicious insulin therapy may be particularly impactful in the COVID-AP interface, where glucocorticoids and vasopressors are frequently co-administered and iatrogenic glycemic excursions are common [96]. In our study, we found higher serum glucose in patients with AP, with similar values for HbA1c in both groups and with a good predictive value for serum glucose in patients with AP (AUC = 0.633, *p* = 0.012), suggesting that serum glucose can be considered a potential biomarker in COVID-19-associated AP.

Severity of lung injury in COVID-19 appears to be a clinically meaningful amplifier of risk for pancreatic injury and for worse trajectories in established AP. In patients with SARS-CoV-2, progressive alveolo-capillary damage, immunothrombosis, and endotheliitis promote a high-grade systemic inflammatory and pro-thrombotic state that perturbs pancreatic microcirculation, lowers the threshold for ischemia–reperfusion injury, and intensifies necro-inflammation once AP is triggered. Autopsy studies demonstrate diffuse pulmonary microvascular thrombosis and angiogenesis—pathologies that correlate with severe hypoxemia and reflect generalized endothelial activation in COVID-19 [98]. Zuo et al.showed that NETs, elevated with increasing COVID-19 severity, promote thrombo-inflammation and microvascular occlusion, providing a mechanistic bridge from lung injury to distal organ ischemia [83]. Lung injury in COVID-19 intersects with well-described determinants of AP severity. Microcirculatory failure—characterized by capillary no-reflow, leukocyte–endothelial adhesion, hemoconcentration, and microthrombosis—is a central pathological axis in severe AP and renders the pancreas exquisitely vulnerable to systemic hypoxemia and endothelial dysfunction [99,100].

Severe lung injury may heighten AP risk through three convergent pathways. Firstly, hypoxemic respiratory failure reduces pancreatic oxygen delivery, amplifying ischemia–reperfusion-driven acinar injury in a gland already prone to perfusion defects [99,100]. Secondly, “biotrauma” from ventilator-induced lung injury and acute respiratory distress syndrome (ARDS) triggers systemic cytokine storm, which can induce organ apoptosis and endothelial damage, including in the splanchnic vascular bed [101,102]. Thirdly, COVID-19-related immunothrombosis (NETs, platelet activation) induces microthrombi formation within pancreatic capillaries, worsening the no-reflow phenomenon associated with severe AP [83,99,100]. Although current evidence suggests rather than proves direct causality, the biological gradient between lung injury severity and systemic endotheliopathy provides strong plausibility, ensuring future research hypotheses. In our study, we found that COVID-19 patients with AP have more severe interstitial lesions with significantly increased total pulmonary lesions than patients with COVID-19 alone, demonstrating a good independent association (AUC = 0.746, *p* < 0.001);therefore, ithas the potential to be used as a risk biomarker.

Limitations of the study:The single-center and retrospective nature of the study limits itsgeneralizability. The absence of histopathologic confirmation restricts mechanistic clarity. The temporal overlap of biological markers for AP and SARS-CoV-2 makes it difficult to estimate the impact of each disease on markervariation. The study focuses on patients with severe forms of SARS-CoV-2 infection, which can overestimate the prevalence of AP in the general population with SARS-CoV-2 infection. Potential confounding from therapies (e.g., steroids, NSAIDs) that may induce pancreatic injury.

## 5. Conclusions

This study underscores the complex interplay between systemic inflammation, coagulation disturbances, metabolic dysregulation, and organ-specific injury in the development of AP among COVID-19 patients. Biomarkers, including LDH, ESR, CRP, fibrinogen, D-dimers, and elevated neutrophil counts, reflect hyperinflammatory and pro-thrombotic states that may predispose to pancreatic injury. Concurrent hypokalemia, stress hyperglycemia, and severe pulmonary involvement further exacerbate disease severity through ischemia, endothelial dysfunction, and necro-inflammatory cascades. These findings suggest that integrated assessments of inflammatory, hematologic, metabolic, and pulmonary parameters may facilitate early identification and risk stratification of COVID-19 patients susceptible to AP. Although AP is not an independent predictor of mortality, early identification of pancreatic injury in severe COVID-19 patients remains essential, as it is associated with greater disease severity, prolonged hospitalization, and an increased risk of organ failure.

## Figures and Tables

**Table 1:** Technical parameters of CT scans.

Parameters	CT Scan Values
Slice thickness (mm)	3
Reconstruction thickness (mm)	1.5
Colimmation	1.2
Reference mAs	250
Reference kV	120
Rotation time (s)	0.5
Pitch	0.35
FOV	Both lungs/thorax and superior abdomen included
Reconstruction kernels	H31f for mediastinum and superior abdomen; H60f for lung

Abbreviations: mAs—milliangstroms, kV—kilovolts, FOV—field of view; mm—milimeters.

**Table 2:** Comorbidities in SARS-CoV-2 patients with and without AP.

Comorbidity	Group A (*n*, %)	Group B (*n*, %)
Obesity	14 (43.7%)	126 (33.7%)
Diabetes mellitus type-2	7 (21.8%)	36 (9.6%)
Arterial hypertension	17 (53.1%)	115 (30.8%)
Congestive heart failure	2 (6.2%)	7 (1.8%)
Peripheral vascular disease	1 (3.1%)	4 (1.1%)
Chronic obstructive pulmonary disorder	2 (6.2%)	25 (6.7%)
Chronic viral hepatitis	1 (3.1%)	11 (2.9%)
History of neoplasia	2 (6.2%)	14 (3.7%)
History of ischemic stroke	1 (3.1%)	18 (4.8%)
Dementia	1 (3.1%)	5 (1.3%)
History of peptic ulcer	1 (3.1%)	4 (1.1%)

**Table 3:** Clinical, biological, and imaging characteristics in Group A and Group B patients.

Clinical, Biological, and Imaging Characteristics	Group A (Median, Q1, Q3)	Group B (Median, Q1, Q3)	*p*-Value
Heart rate (beats/min)	96.5 [82.5; 111.2]	93 [81; 106]	0.174
Respiratory rate (breaths/minute)	17 [16; 21]	18 [16; 20]	0.776
Saturation (O_2_) %	92 [88.2; 96.7]	92 [86; 96]	0.694
Hospital stay (days)	15 [11; 23]	11 [6.75; 18.2]	0.003
Duration of antiviral treatment (days)	5.5 [4.2; 9.7]	4 [3; 5]	0.001
Duration of corticotherapy(days)	10 [7; 16.7]	7 [1; 12]	0.005
Serum glucose (mg/dL)	122.5 [109.5; 142]	112 [100; 129]	0.012
HbA1c %	5.9 [5.3; 6.6]	5.8 [5.3; 6.6]	0.816
CRP (mg/L)	92.3 [38.2; 140.4]	39 [12;73.1]	<0.001
Fibrinogen (mg/dL)	635 [419.5; 708.5]	447 [335.5; 562]	0.001
ESR(mm/h)	59 [52; 69]	45 [20; 55]	<0.001
LDH (U/L)	381 [308.5; 456.7]	257 [216; 330]	<0.001
AST (U/L)	46 [38.5; 65]	38 [30; 53.7]	0.002
ALT (U/L)	43 [30.2; 54]	38 [24; 54]	0.331
CK (U/L)	107 [50.7; 232.7]	104 [48; 148]	0.271
Serum lipase (U/L)	552.5 [462.5; 761.2]	124 [84; 195.5]	<0.001
Serum urea (mg/dL)	31.4 [28.6; 40.6]	32.6 [26; 40.5]	0.667
Serum creatinine (mg/dL)	0.8 [0.7; 1]	0.8 [0.6; 0.9]	0.206
DB (mg/dL)	0.3 [0.2; 0.5]	0.4 [0.3; 0.5]	0.220
IB (mg/dL)	0.4 [0.3; 0.4]	0.5 [0.3; 0.6]	0.673
Serum ferritin(ng/mL)	715 [493; 1163]	633 [311.2; 1134.2]	0.234
IL1 (pg/mL)	0.3 [0.1; 3.5]	5.7 [0.5; 18.7]	0.024
IL6 (pg/mL)	107.2 [45.2; 164.4]	91.4 [36.2; 149.1]	0.418
TNFα (pg/mL)	9.4 [3.3; 17.5]	10.7 [4.8; 18.3]	0.636
RBC (×10^6^/µL)	4.5 [3.9; 4.8]	4.4 [3.9; 4.9]	0.859
WBC (×10^3^/µL)	8.7 [6.9; 12.8]	6.4 [5; 9.4]	0.001
Lymphocytes (×10^3^/µL)	0.9 [0.5; 1.1]	0.9 [0.6; 1.4]	0.341
Neutrophils (×10^3^/µL)	7.2 [5.9; 11]	4.5 [3.2; 7.3]	<0.001
Platelets (×10^3^/µL)	232 [159.5; 332.5]	188 [138; 232.5]	0.028
D-dimers (ng/mL)	264 [193.5; 487]	187.5 [144; 259]	0.001
Serum Na (mEq/L)	138 [132.2; 159.5]	137 [130; 142]	0.123
Serum K (mEq/L)	4.5 [3.9; 5.2]	4.2 [3.7; 4.6]	0.021
Number of pulmonary lobes involved	5 [5; 5]	5 [4; 5]	0.008
Consolidation (% from total lung volume)	1 [0.7; 1.7]	0.9 [0.7; 1.6]	0.526
Mixed lesions (% from total lung volume)	2.9 [2.6; 4.9]	2.4 [1.2; 3.8]	0.005
Interstitial lesions (% from total lung volume)	42.1 [33.9; 54.6]	30.3 [21.5; 43.4]	<0.001
Normal pulmonary densities (% from total lung volume)	48.7 [34.6; 56.9]	61.4 [47.2; 69.5]	<0.001
Total pulmonary lesions (% from total lung volume)	47.4 [37.9; 63.4]	34.4 [23.6; 49.1]	<0.001

Abbreviations: ALT—alanine transaminase; AST—aspartate transaminase; CK—creatine kinase; CRP—C-reactive protein; DB—direct bilirubin; ESR—erythrocyte sedimentation rate; HbA1c—glycated hemoglobin; IB—indirect bilirubin; IL—interleukin; K—potassium; LDH—lactate dehydrogenase; Ly—lymphocytes; Na—natrium/sodium; Neu—neutrophils; Q1—first quartile; Q3—third quartile; RBC—red blood cells; TNFα—tumor necrosis factor alpha; WBC—white blood cells.

**Table 4:** Characteristics of AP in Group A patients.

Parameter		Patients in Wave 1	Patients in Wave 2	Patients in Wave 3
Balthazar grade	0	0	0	0
	1	4	5	3
	2	4	3	5
	3	2	1	4
	4	0	0	1
Pancreaticnecrosisscore	0%	9	9	11
	≤30%	1	0	2
	30–50%	0	0	0
	50%	0	0	0
CTSI	0–3	9	9	11
	4–6	1	0	2
	7–10	0	0	0
Lipase level	normal	0	0	0
	1–3×	7	8	10
	>3×	3	1	3

**Table 5:** Correlations of clinical, biological, and imaging characteristics with AP.

Clinical, Biological, and Imaging Characteristics	Spearman’s Rho	*p*-Value
Hospital stay	0.163	0.003
Duration of antiviral treatment (days)	0.179	0.001
Duration of corticotherapy	0.148	0.005
Serum glucose	0.124	0.012
CRP	0.197	<0.001
Fibrinogen	0.164	0.001
ESR	0.259	<0.001
LDH	0.290	<0.001
AST	0.154	0.002
IL1	−0.164	0.023
WBC	0.159	0.001
Neutrophils	0.193	<0.001
Platelets	0.109	0.028
D-dimers	0.172	0.001
Serum K	0.116	0.020
Number of pulmonary lobes involved	0.161	0.001
Mixed lesions (% from total lung volume)	0.140	0.005
Interstitial lesions (% from total lung volume)	0.230	<0.001
Normal pulmonary densities (% from total lung volume)	−0.211	<0.001
Total pulmonary lesions (% from total lung volume)	0.217	<0.001

Abbreviations: AST—aspartate transaminase; CRP—C-reactive protein; ESR—erythrocyte sedimentation rate; IL—interleukin; K—potassium; LDH—lactate dehydrogenase; WBC—white blood cells.

**Table 6:** ROC curve analysis for parameters associated with AP.

Predictor	AUC	Std Error	*p*-Value	CI 95%	
				Lower Bound	Upper Bound
Hospital stay	0.661	0.041	0.003	0.581	0.741
Duration of antiviral treatment (days)	0.677	0.051	0.001	0.576	0.777
Duration of corticotherapy	0.650	0.048	0.005	0.556	0.745
Serum glucose	0.633	0.046	0.012	0.542	0.724
CRP	0.702	0.048	<0.001	0.609	0.795
Fibrinogen	0.675	0.057	0.001	0.563	0.787
ESR	0.777	0.033	<0.001	0.711	0.842
LDH	0.806	0.032	<0.001	0.743	0.868
AST	0.662	0.037	0.002	0.589	0.735
IL1	0.695	0.071	0.024	0.556	0.834
WBC	0.670	0.048	0.001	0.575	0.764
Neutrophils	0.707	0.046	<0.001	0.618	0.796
Platelets	0.617	0.055	0.028	0.509	0.725
D-dimers	0.684	0.057	0.001	0.573	0.795
Serum K	0.623	0.058	0.021	0.510	0.736
Number of pulmonary lobes involved	0.649	0.041	0.005	0.568	0.730
Mixed lesions (% from total lung volume)	0.652	0.040	0.005	0.572	0.727
Interstitial lesions (% from total lung volume)	0.746	0.036	<0.001	0.675	0.818
Normal pulmonary densities (% from total lung volume)	0.726	0.035	<0.001	0.657	0.795
Total pulmonary lesions (% from total lung volume)	0.733	0.036	<0.001	0.663	0.802

Abbreviations: AST—aspartate transaminase; CRP—C-reactive protein; ESR—erythrocyte sedimentation rate; IL—interleukin; K—potassium; LDH—lactate dehydrogenase; WBC—white blood cells.

**Table 7:** Multivariable logistic regression model for patients with AP.

Variable	B	S.E.	Wald	*p*	OR	95% CI for OR	
						Lower	Upper
D-Dimers	0.002	0.001	7.054	0.008	1.002	1.000	1.003
Lung interstitial lesions	0.045	0.017	7.355	0.007	1.046	1.013	1.081
CRP	0.011	0.003	13.083	<0.001	1.011	1.005	1.018
Constant	−5.552	0.820	45.808	<0.001			

Abbreviations: CI—confidence interval; CRP—C-reactive protein; OR—odds ratio; S.E.—standard error.

## Data Availability

The original contributions presented in this study are included in the article. Further inquiries can be directed to the corresponding author.
